# Triboelectric Energy-Harvesting Floor Tile

**DOI:** 10.3390/ma15248853

**Published:** 2022-12-12

**Authors:** Panu Thainiramit, Subhawat Jayasvasti, Phonexai Yingyong, Songmoung Nandrakwang, Don Isarakorn

**Affiliations:** 1 Department of Instrumentation and Control Engineering, School of Engineering, King Mongkut’s Institute of Technology Ladkrabang, Bangkok 10520, Thailand; 2 Department of Industrial Education in Engineering Education, King Mongkut’s Institute of Technology Ladkrabang, Bangkok 10520, Thailand

**Keywords:** triboelectric energy harvesting, triboelectric material thickness, energy harvesting floor tile, impact force, human footsteps

## Abstract

The aim of this study was to investigate the real-world electrical parameters that strongly affected the performance of a triboelectric energy-harvesting floor tile design: triboelectric material thickness, cover plate displacement distance or gap width, and cover plate pressing frequency, so that real-world specifications of the harvesting floor tile can be accurately specified. The structure of the designed triboelectric energy harvester, with readily available polytetrafluoroethylene (PTFE) film and aluminum foil, was simple and hence easy to fabricate, and the material cost was low. A square wave was used to simulate the pressing frequency on the test bench’s cover plate. The results showed that the voltage and current were proportional to the gap width, and the thinner the triboelectric layer thickness, the higher the output voltage and current. A test bench with a 0.2 mm thick PTFE triboelectric layer generated the highest energy output. In a later experiment, a triboelectric energy-harvesting floor tile (TEHFT) prototype was constructed with 0.1 and 0.2 mm thick PTFE layers. We found that at 2 Hz stepping frequency and 0.1 mm PTFE thickness, the optimal load and cumulative energy of the TEHFT were 0.8 MΩ and 3.81 mJ, respectively, while with 0.2 mm PTFE thickness, these two parameters were 1.1 MΩ and 7.69 mJ, respectively. The TEHFT with 0.2 mm thick PTFE layer was able to illuminate a series of 100 to 150 LEDs, sufficient power to drive small electronics and sensor nodes. This discovery provides important data on the structure, material, and contact surface area of a TEHFT that can be adjusted to suit specific requirements of a special function triboelectric energy harvester.

## 1. Introduction

Kinetic energy from the ambient environment can be an energy source that provides high-power density to power small electronic devices as a substitute to a battery, economizing energy usage and reducing heavy-metal pollution. Human footsteps are an important and sustainable energy source to investigate because of the high-power density of the ever-growing population and places to walk along. The favored methods for converting impact force from footsteps to electrical energy were piezoelectric [1], electromagnetic [2], and triboelectric [3], installed in the insole of shoes and fabricated floor tile [4,5]. As previously shown in [6,7], a piezoelectric test bench and a piezoelectric energy harvesting floor tile for real-world application were constructed. Panthongsy et al. [8] developed a test bench for harvesting energy from a mechanical vibration source, using a piezoelectric cantilever to convert mechanical vibration energy into electrical energy. The test bench was based on frequency up-conversion for high performance, which was then constructed into a prototype of energy harvesting floor tile. Later, Yingyong et al. [9] investigated the electrical performance of an energy-harvesting floor tile based on double-stage frequency up-conversion under a laboratory test and a field test. The human walking parameters were investigated for the field test, including the pedestrian weight, human pace (walking and running), and pedestrian density. The results showed that the impact force from human weight and the pedestrian density did not affect the electrical output so much, but the impact force from human walking and running influenced the electrical energy of this harvester significantly. Regardless, all investigations showed that the harvester prototype was able to provide sufficient energy for real-world application. However, even though the piezoelectric energy harvester could generate high output energy, the electrical output density per the cost of energy harvesting material was a little high for commercial application. Therefore, other harvesting methods with cheaper material and good power performance were considered. Triboelectric energy harvester was conceptualized because it has a simple structure hence easy to fabricate, and the cost of material is low. A triboelectric energy harvester converts kinetic energy to electrical energy by contacting or sliding two different triboelectric materials against each other. The conversion is based on two mechanisms: contact electrification and electrostatic induction. Triboelectric energy harvester (TEH) is classified into four modes: vertical contact-separation (CS), lateral-sliding (LS), single-electrode (SE), and freestanding triboelectric-layer (FT) [10,11,12]. Of the four modes, vertical contact-separation was the most interesting and appropriate to investigate as a waste energy harvester from human motion such as human footsteps. A research work [13] investigated a piezoelectric and a triboelectric energy harvester by constructing test benches. The cover plate of each was repeatedly pressed and released to activate. Then, the power density and energy density of those two different test benches were compared. The results showed that piezoelectric energy harvester (PEH) test bench was able to generate higher output power density and energy density than triboelectric energy harvester (TEH) test bench, but the cost of the piezoelectric material was way higher than that of the triboelectric material. TEH has gained much attention in many applications and is an increasing trend for publication [14,15,16,17]. Niu and Wang [18] summarized the basic theory of triboelectric energy harvester, including the working principle of triboelectric energy harvester, the relationship equation between three parameters: output voltage, transfer charge, and mechanical displacement. Several parameters influenced the electrical performance of TEH, e.g., the air gap between two different triboelectric materials, the material properties, and the environmental condition. Zhang et al. [19] investigated the influence of applied force and dielectric thickness on the output performance of a TEH, and they fabricated a dielectric-to-dielectric contact-separation mode triboelectric energy harvester) to harvest energy from impact force. The results showed that the thickness of the triboelectric Polytetrafluoroethylene (PTFE) sheet affected the electrical output of the TEH significantly; an optimal PTFE layer thickness was able to generate high output signal and transferred charge. Gomes et al. [20] investigated the effect of triboelectric material thickness and contact surface area on the performance of a TEH. The results showed that the output voltage decreased when the dielectric thickness increased, but the output current increased. When calculated for output power, it would reach the maximum power at an optimal thickness. The basic theory and several papers mentioned above did not conclude about the effective thickness; therefore, we could not choose a proper thickness of dielectric material for fabricating a high performance TEHFT for this study. Hence, this work aimed to investigate the effect of PTFE thickness on the electrical performance of a TEH test bench (TEHTB). Moreover, in the TEHTB fabrication, the gap between the cover plate and the base was designed not to be too far apart for people to step on comfortably. Even though the farther the gap width, the higher the cover plate acceleration and the higher the output voltage, if the gap width was too wide for comfortable stepping, it would not be practical. In addition, we aimed to investigate another important parameter for practical energy harvesting from human footsteps: human stepping frequency. Several other studies have investigated the pressing frequency of an external force (e.g., footstep) on the cover plate of a TEH test bench. However, the experimental results varied; some studies found that the pressing frequency affected the amplitude of the TEH test bench’s electrical signal significantly, while others did not. For example, Oh et al. [21] developed a porous non-woven thermoplastic polyurethane/polypropylene triboelectric nanogenerator to harvest human walking energy. The triboelectric nanogenerator was fabricated in the insole of footwear. It operated in contact-separation mode. The results from applying a fixed external force with varying frequencies (from 0.5 to 8 Hz) showed that the open-circuit voltage and short circuit current tended to increase with increasing pressing frequency, which corresponded well with the results of a study by Xia et al. [22]. As another example, Saadatnia et al. [23] developed a contact-separation TEHTB with porous polyimide aerogel film as a triboelectric material. The effect of excitation frequency (from 5 to 8 Hz) on the open-circuit voltage of the TEHTB was investigated. The results showed that the output voltage amplitudes at any tested excitation frequencies were almost the same. Palaniappan et al. [24] investigated triboelectric nanogenerators using the contact-separation mode, using two different triboelectric materials. The negative triboelectric layer was Kapton, while the positive triboelectric layer was Polydimethylsiloxane (PDMS). They investigated its electrical characteristics and applied an external force at the frequency from 5 Hz to 40 Hz; the generated open circuit voltage was stable at 4 V_P-P_. Most research works mentioned above investigated high pressing frequency, contact-separation mode TEH, but the normal human walking frequency was about 2 Hz [25,26]. Walking frequency over an area varies with pedestrian density and demography. Stepping frequency on a floor tile might vary, and it would affect the electrical output of the TEHFT significantly. Therefore, to construct a high-performance TEHFT that truly suited to human footsteps, it is necessary to obtain more information on the electrical behavior of the TEHTB. This work aimed to investigate the effect of triboelectric material thickness and contact displacement (gap width) on the electrical characteristics of a TEHTB. The triboelectric materials that were used followed the trend of triboelectric series [11]. In the triboelectric series, there are lists of two different materials’ properties: positively charged material and negatively charged material. The longer the separation in the list between two triboelectric materials in a triboelectric series, the greater surface charge density during the contact electrification process. Negatively charged substances, such as PTFE, PDMS, and fluorinated ethylene propylene (FEP), were commonly employed. Other materials, including Kapton, polyethylene terephthalate (PET), and silicone, were also exploited, but their lower charge affinities make them less commonly used than those listed above. Zhang et al. [27] summarized the triboelectric material choices from 100 random articles. PTFE was used as a triboelectric layer for about 34% with 14 different electron acceptor materials. Aluminum (Al) was used as a positive triboelectric layer and electrode for about 26% with 20 different electron donor materials. Due to the trend and common use of triboelectric material in several research studies, PTFE film and Al were the choices for the dielectric-to-conductor TEHTB in this work. In addition, we also investigated the influence of excitation frequency on the electrical output of the TEHTB since this parameter was crucial for predicting the cumulative energy created by a TEHFT in practical applications. In addition, when the appropriate thickness of triboelectric material in the TEHTB was known, it was used for the fabrication of the TEHFT prototype. The electrical characteristics and practical application of TEHFT were also investigated. We believe that the outcomes of this study will benefit actual applications and provide more crucial information for the future development of high-performance TEHFTs.

The rest of this paper is structured as follows: Section 2 describes the verification of a TEHTB mechanism’s design feasibility, including the prototype of the TEHTB, verification method, and results; Section 3 presents an evaluation of the TEHFT prototype, including its structural components and electrical characteristics, with a proper triboelectric material thickness, and discussion of the practical application of the TEHFT prototype; lastly, a summarization of the study is presented in Section 4.

## 2. Performance Evaluation of TEHTB

### 2.1. TEHTB Structure and Working Principle

**Figure 1:**
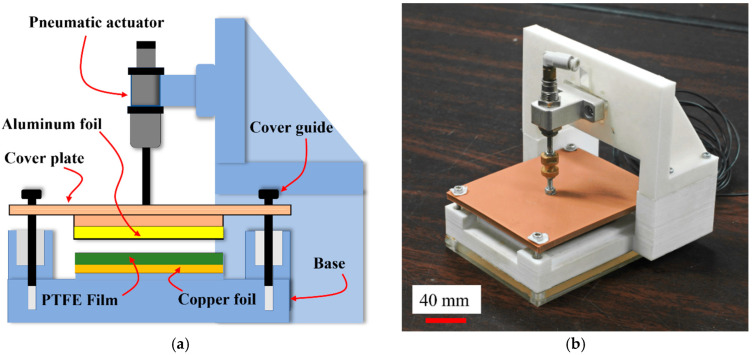
TEHTB structure: (**a**) essential component: cover plate, cover guide, triboelectric material (PTFE film), Aluminum foil (top electrode), and copper foil (bottom electrode); and (**b**) photograph of TEHTB.

In this study, the triboelectric energy-harvester test bench (TEHTB) structure works in contact-separation mode. A dielectric triboelectric material with copper foil electrode backing is pushed into contact with aluminum foil, as shown in Figure 1.

**Figure 2:**
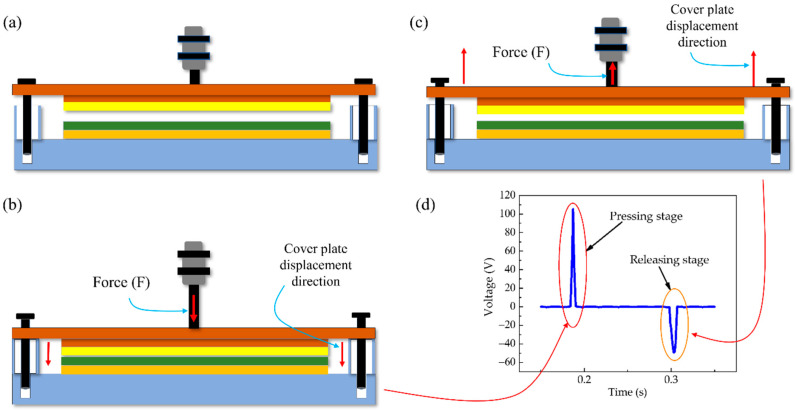
TEHTB working operation: (**a**) standstill stage: no output voltage; (**b**) pressing stage: force direction and cover plate displacement direction; (**c**) releasing stage; and (**d**) output voltage graph.

The test bench was designed to harvest energy from the impact force of an external energy source. There were two main parts: the cover plate and the base. We experimented with aluminum foil (50 mm × 50 mm × 0.022 mm) as the top electrode, attached to the underside of the cover plate, and 8 thicknesses (0.1, 0.2, 0.3, 0.4, 0.5, 0.8 and 1 mm) of triboelectric Polytetrafluoroethylene (PTFE) sheet, attached to a copper foil (50 mm × 50 mm × 0.05 mm) electrode, both layers mounted on top of the base of the test bench. The cover plate was attached to the pneumatic actuator with a nut and bolt. The actuator pushed down on the cover plate with an intended impact force and pulled it up away from the base by the force of the spring in the actuator when it was deactivated. In the TEHFT prototype, the return springs were alongside the cover guides. Linear guides were used to restrict the movement of the cover plate to a strictly vertical direction. When the aluminum foil electrode came into contact-separation with the triboelectric material, voltage difference was generated across the aluminum foil electrode and the triboelectric PTFE layer, as shown in Figure 2.

### 2.2. Experimental Method

This section presents an overview of the experimental setup of TEHTB. The setup allowed for adjustable mechanical energy input and pressing frequency on the cover plate. The details of the experiment and procedures are described as follows.

**Figure 3:**
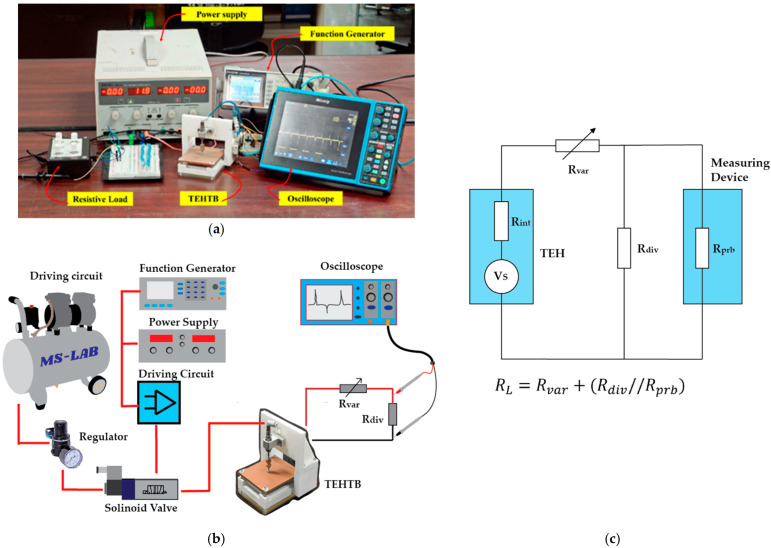
(**a**) The experiment setup for the TEHTB; (**b**) the schematic of the experimental setup; and (**c**) the schematic diagram of the measurement technique.

The experiment was to apply mechanical input from a pneumatic actuator as an impact force. A pneumatic actuator (CJPB6-15 from SMC Corporation) provided the pushing force of 13.04 N at the operation pressure of 0.6 MPa controlled by regulator and the pulling force of 1.42 N by its return spring. The accelerated movement of the cover plate was measured with an accelerometer (EI-CALC). The pressing frequency of the pneumatic actuator was controlled by a square wave signal from a function generator (GW INSTEK AFG-2225). An oscilloscope (Micsig, model STO1104C) with a probe resistance of 1 MΩ measured the electrical output at room temperature of 25 °C and relative humidity of about 70% in the laboratory, Thailand. A photo of the experiment setup for the TEHTB is shown in Figure 3a, while the schematic of the experiment setup is illustrated in Figure 3b.

On a practical measurement issue, the TEHTB is a high-internal-resistance energy harvester, higher than the probe resistance of a typical oscilloscope. Therefore, a conventional measurement technique could not indicate its maximum power transfer, so we used a voltage divider to measure the output voltage across the total external resistive load and Equation (1). Instead of measuring the voltage across the TEHTB, the voltage across an introduced, parallel-connected resistor divider was measured using an oscilloscope, as shown in Figure 3c. For more details about this technique, see reference [28]. The resistance ratio between the resistor divider and probe resistance was 1:10 as recommended in the reference, and the resistor divider in this study was fixed at 0.1 MΩ. Figure 3c showed the schematic diagram of the measurement technique for the high internal resistance of the TEHTB, where $V_{s}$ was the source voltage; $R_{int}$ was the internal resistive load of the TEH; $R_{var}$ was the variable resistive load; $R_{div}$ was the voltage divider resistive load; $R_{prb}$ was the probe resistance of the measuring device; $V_{read}$ was the actual voltage measured by the oscilloscope; and $V_{L}$ represented the theoretical voltage produced by the TEHTB across the load resistor, $R_{L}$, $R_{L}=R_{var}+\left(R_{div}//R_{prb}\right)$, where

(1) $$V_{L}=\frac{V_{read}\times R_{L}}{\left(R_{div}/\;/R_{prb}\right)}$$

The energy generated by the TEHTB was an essential electrical characteristic for evaluating its performance and application. Generally, the energy produced by the energy harvester can be calculated by the voltage across the capacitor. One problem of energy evaluation by charging a capacitive load is that leakage current will need to be monitored. Low leakage current is especially important when the energy source delivers low currents that are not significantly higher than the low leakage current itself. For this reason, we chose to use transient analysis to calculate the energy generated by the TEHTB. As indicated in the following equation, the energy produced at the time $t_{N}$ could be determined using Equation (2) [29], where time $t_{n}$ was the time at sub-step n; $V_{L}(t_{n})$ was the voltage across the resistor at the same sub-step at the time instant *t_n_*; and $\Delta t_{n}$ was the duration of each sub-step,

(2) $$E\left(t_{N}\right)=\sum_{n=0}^{N}\frac{V_{L}^{2}\left(t_{n}\right)}{R_{L}}\Delta t_{n}\;\;\;\;\;\;\;\;\;\;\;\;\mathrm{For}\;N\;>\;0.$$

The procedural steps in the experiment were as follows:

(1) Twenty repeatedly measured voltages were obtained for the evaluation; current and power were derived from the obtained voltage.
(2) Various thicknesses of the PTFE sheet inside the test bench were electrically tested; the displacement (gap width) between the cover plate and the base was varied to be 2, 4, 6, 8, and 10 mm.
(3) The effect of pressing frequency on the electrical output of the TEHTB was examined by varying the pressing frequency on the cover plate from 0.5 to 3 Hz.
(4) The electrical outputs of the TEHTB with different PTFE thicknesses were compared, then two optimal thicknesses were selected for fabricating the TEHFT prototype.

It should be noted that all electrical parameters, i.e., voltage, current, and power are the instantaneous values. 

### 2.3. TEHTB Experimental Results and Discussion

Electrical properties of the TEHTB were investigated in comparing various thicknesses of the triboelectric PTFE layer, and the optimal thicknesses for TEHFT prototype fabrication were determined. The pneumatic pressure was kept constant while the displacements at 2, 4, 6, 8, and 10 mm, were tested; the root mean square (RMS) acceleration of the cover plate, at the moment of contact, was 0.41 g, 0.64 g, 0.7 g, 0.8 g, and 1.08 g, respectively when the fixed external force was applied. These accelerated motion values were within the gait acceleration (RMS) range reported by Satkunskiené et al. [30] and the gait acceleration (peak) range reported by Morrow et al. [31]. A heavier person stepping on the TEHFT prototype would result in greater acceleration than a lighter person, and a person running would result in greater acceleration than walking [8].

**Figure 4:**
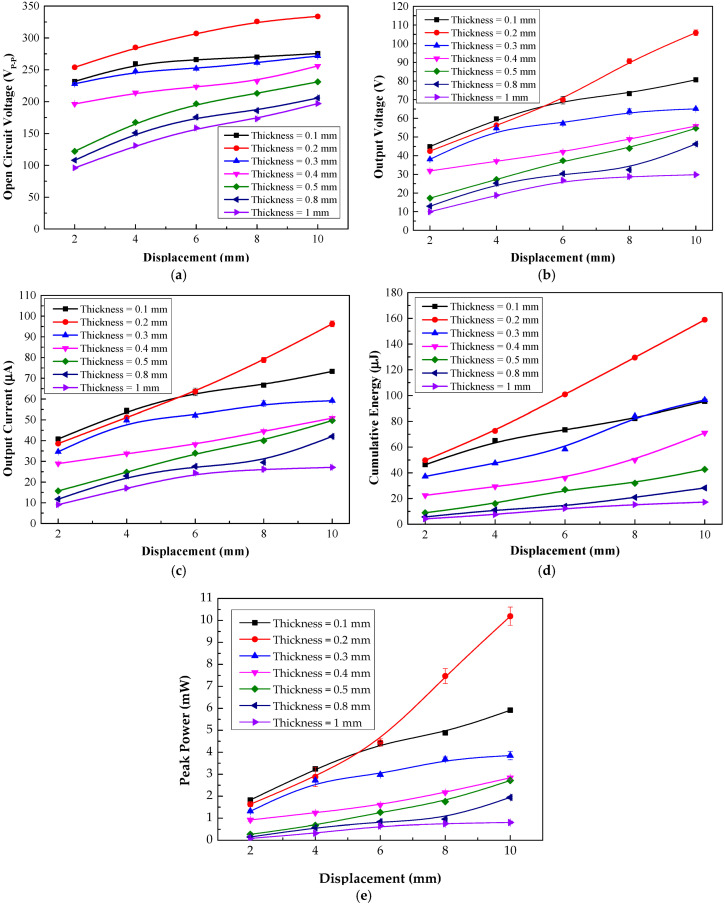
Electrical characteristics comparison of TEHTB with various PTFE sheet thicknesses under different displacement (gap width) values: (**a**) peak-to-peak open-circuit voltage comparison; (**b**) peak voltage across an external resistive load of 1 MΩ; (**c**) peak currents through an external resistive load of 1 MΩ; (**d**) cumulative energy comparison across a resistive load of 1 MΩ; and (**e**) peak power comparison.

As presented in Figure 4a, the open-circuit voltage of the TEHTB equipped with each PTFE film thickness varied with the cover plate displacement (gap width) in full agreement with the open-circuit voltage equation in the triboelectric theory given in [11,12,13]. The open-circuit output values were compared (peak to peak voltage). A thinner PTFE layer created a higher voltage than a thicker layer. Nevertheless, this experiment revealed that a PTFE film thickness of 0.2 mm was able to develop the highest open-circuit voltage. The highest voltage was 254.0 V at a displacement of 2 mm and climbed to 333.8 V at a displacement of 10 mm. The highest output voltage when connected to a load was achieved by the 0.1 mm thick PTFE, but it did not provide the highest open-circuit voltage. At 2 mm displacement, the open-circuit voltage of TEHTB with a 0.1 mm thin PTFE sheet was 231.8 V, whereas, at 10 mm displacement, it was 275.6 V. With 1 mm PTFE thickness, the TEHTB created the lowest voltage; it produced 96.0 V at 2 mm displacement and 197.0 V at 10 mm displacement.

For the electrical output comparison of two parameters—PTFE film thickness and displacement distance (gap width between the cover plate and base)—a resistive load connected between the two electrodes was fixed at 1 MΩ. Electrical characteristics of the TEHTB with PTFE films of different thicknesses were measured and compared. For measuring the output voltage of TEHTB configured with different displacement distances (gap widths), the resistive load was connected to the TEHTB. Figure 4b shows the relationships between the output voltage, the cover plate displacement distance, and the triboelectric material thickness. Seven different PTFE thicknesses were employed: 0.1, 0.2, 0.3, 0.4, 0.5, 0.8, and 1 mm. According to the triboelectric theory [32], for a fixed value of displacement distance, the thinner the PTFE triboelectric layer is, the higher the output voltage is. The TEHTB demonstrated that this conclusion was valid at a displacement distance of 2 mm and 4 mm, and so we configured our TEHFT prototype accordingly. The peak voltage of a 0.1 mm thin film elevated from 44.86 V at 2 mm displacement to 59.73 V at 4 mm displacement.

The TEHTB with a 0.1 mm thick PTFE layer produced the maximum voltage at a small cover plate displacement distance between 2 mm and 4 mm, but its output voltage was lower than that of the TEHTB with a 0.2 mm thick PTFE layer at any displacement distance greater than 4 mm. We concluded that a PTFE sheet thickness of 0.1 mm would be suitable for energy harvesters that need small displacement distance. At 2 mm and 4 mm displacement distances, the TEHTB with a 0.2 mm thick PTFE layer yielded a lower peak voltage than that with 0.1 mm thick PTFE layer. However, at 6 mm displacement distance, the voltage increased dramatically from 44.42 V at 2 mm displacement to 105.88 V at 10 mm displacement. Based on these test findings, a larger displacement distance can result in a greater output voltage, and a 0.2 mm thick PTFE layer is suitable for use in energy harvesters with a displacement distance greater than 4 mm.

Output current through resistive load was another important electrical property to consider. As illustrated in Figure 4c, the current through a resistive load is proportional to the voltage. The trend of the current comparison graph was identical to that of the voltage comparison graph. The TEHTB with a thinner PTFE sheet delivered a greater current than the one with a thicker sheet at 2 mm and 4 mm displacement distances, providing a maximum current at 40.78 µA and 54.30 µA, respectively. When the displacement was more than 4 mm, the TEHTB with a 0.2 mm thick PTFE layer delivered a greater current than any other thickness.

**Figure 5:**
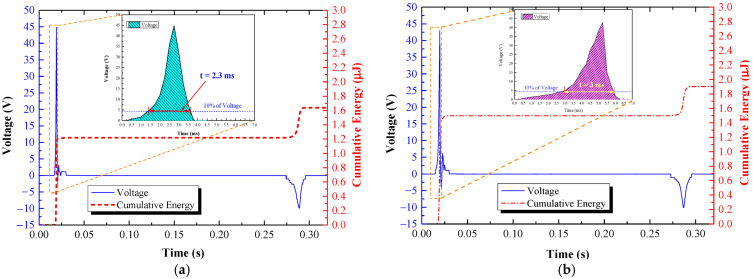
Output voltage and energy curve of TEHTB: (**a**) 0.1 mm thick PTFE layer; (**b**) 0.2 mm thick PTFE layer.

In addition to voltage and current, cumulative energy was an essential electrical attribute for developing an energy harvester. Figure 4d shows a comparison between cumulative energies delivered by TEHTB with various PTFE layer thicknesses. The cumulative energy provided by the TEHTB decreased as the thickness of the PTFE film increased, and the cumulative energy delivered by TEHTB with any PTFE thickness tended to increase with increasing displacement distance. Figure 4e also shows a comparison between peak powers calculated from the output voltages at a resistive load of 1 MΩ. Figure 4 shows that for a short displacement distance between 2 mm to 4 mm, which was required for our developed Triboelectric Energy Harvesting Floor Tile (TEHFT) prototype, the 0.1 mm thick PTFE layer was optimal, while for a longer displacement distance 4 mm to 10 mm, applicable to other mechanical configurations of a potential triboelectric harvester, a PTFE layer of 0.2 mm would be more optimal. The TEHTB with a PTFE thickness of 0.2 mm and 0.1 mm provided high energy at any displacement distance. The TEHTB with 0.1 mm thick PTFE layer generated 46.32 µJ of energy at a displacement distance of 2 mm, and elevated to 95.43 µJ at 10 mm displacement distance. The TEHTB with a PTFE layer thickness of 0.2 mm at any displacement distance generated the highest cumulative energy, which was what the TEHFT prototype was designed for, in addition to a high output voltage, to power small electronic devices. The energy increased dramatically from 49.73 µJ at a displacement distance of 2 mm to 158.85 µJ at a displacement distance of 10 mm; hence, if the operational requirement allows a triboelectric energy harvester that can accommodate a higher displacement distance of up to at least 10 mm between the triboelectric layer and the positive electrode will certainly provide a higher cumulative energy. Regarding the TEHFT prototype, it was constructed with both 0.1 mm and 0.2 mm thick PTFE layer because the former provided the highest voltage output, and the latter provided the highest cumulative energy. We wanted to check out which one was the most suitable as an energy harvesting floor tile. An explanation of why 0.1 mm thick PTFE layer provided higher output voltage and current but lower cumulative energy is illustrated in Figure 5. It should be noted that the cumulative energy was calculated from Equation (2). The electrical characteristics of our TEHTB followed the same trend as those of a triboelectric harvester introduced by Gomes et al. [20], but the exact triboelectric layer thickness and displacement distance were different. Similarly, the electrical characteristics of our TEHTB follow the same output voltage trend as the energy harvester, with nylon-PTFE triboelectric layer in contact-separation mode, introduced by Zhang et al. [19], but ours provides higher output voltage.

**Figure 6:**
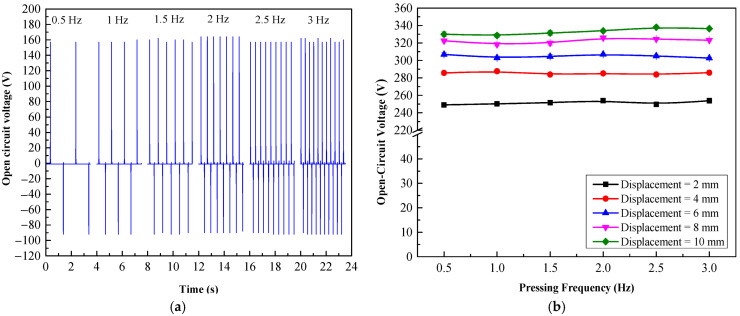
Effect of pressing frequency on the open-circuit voltage of TEHTB with 0.2 mm thick PTFE layer: (**a**) the open-circuit voltage at a displacement distance of 2 mm; (**b**) peak-to-peak voltage comparison while varying displacement distances at different pressing frequencies.

Another important parameter for harvesting energy from human footsteps was their pressing or stepping frequency. It could affect the amplitude of the output voltage of the TEHTB. We realized that the stepping behavior on a floor tile was a stepping on and stepping off action. Therefore, the effect of stepping frequency on the output voltage was simulated by using a square wave signal to control the pressing frequency on the TEHTB. Figure 6a displayed the open-circuit voltage curve for the pressing frequency range of 0.5 Hz to 3 Hz; the voltage was steady at all pressing frequencies and followed the same pattern for all displacement distances, as can be seen in Figure 6b. The voltage was steady, and only the number of voltage cycles increased proportionally to the pressing frequency, causing cumulative energy to increase as the pressing frequency varied. The amplitude of output voltage spikes of the TEHB with different PTFE thicknesses followed the same trend regardless of pressing frequency. The findings from this investigation corresponded with those of Zang et al. [19]; they applied a force at multiple frequencies ranging from 0.5 to 3 Hz, and the output voltages were practically constant at all tested frequencies. Similarly, Palaniappan et al. [24] created a TEH utilizing PDMS and Kapton as triboelectric materials. They increased the pressing frequency on their TEH from 5 to 40 Hz with 5 Hz increments, and the open-circuit voltage remained steady at 4 V because the total number of charges transferred between the triboelectric layers at any given time was constant, the open-circuit voltage remained consistent and stable as the frequency increased. In contrast, if the pressing frequency was simulated as a sine wave, the varying pressing frequency would affect the amplitude of the output voltage, as a study by Yang et al. [33] demonstrated; they investigated the effect of the moving speed of the top electrode on the output performance of a triboelectric energy harvester of which the impact force was controlled by a continuous dynamic sinusoidal waveform from a Keyboard Life Tester (ZX-A03), with varying frequency from 0.5 to 2.5 Hz, and the results showed that the output voltage increased with increasing frequency. Another example that showed that the output voltage was dependent on pressing frequency is a study by Tronco Jurado et al. [34]; they investigated the dielectric-to-conductor contact-separation TEH for harvesting energy from the ocean wave impact, sine wave generated by a function generator was used to control a mechanical shaker to simulate ocean wave impact with oscillating frequencies ranging from 25 and 300 Hz, and found that the peak output voltage fluctuated from 5 V to 20 V. This aspect shows that our energy harvesting floor tile is a good design for its purpose because no matter how frequent it has been stepped on, the output voltage does not vary, and so the floor tile will provide sufficient voltage to power electronic device even though it may be stepped on infrequently. In conclusion, this study explored three crucial factors (triboelectric material thickness, cover plate displacement distance, and cover plate pressing frequency) for an energy harvesting floor tile and found that the optimal triboelectric material thickness was 0.1 and 0.2 mm for a displacement distance between 2 to 4 mm, and cover plate pressing frequency presented no issue with the output voltage. A TEHFT prototype was then constructed with these parameters in consideration. 

## 3. Performance Evaluation of the Triboelectric Energy Harvesting Floor Tile Prototype

### 3.1. Triboelectric Energy Harvesting Floor Tile (TEHFT)

**Figure 7:**
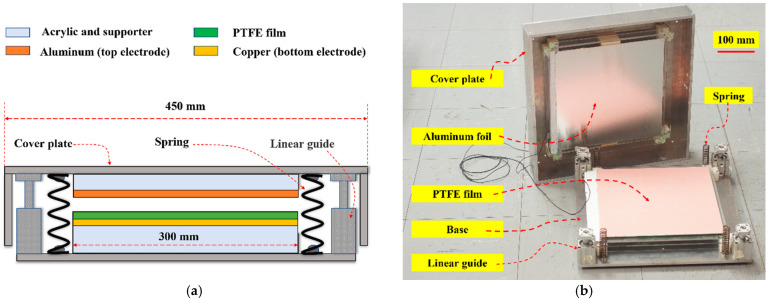
Structural components of TEHFT prototype: (**a**) cross-section; (**b**) photo.

Figure 7 shows the structure of the TEHFT prototype constructed with an aluminum cover plate and a base. The dimensions of the cover plate and the base were 450 mm × 450 mm × 95 mm. The cover plate displacement was 5 mm to get a high harvested cumulative energy but still comfortable enough to step on. The contact surface area for the triboelectric layer was 300 mm × 300 mm, as large as could be fitted to the base area that also had to house the springs and linear guides. 

The top triboelectric material and electrode (Aluminum foil) was attached to an acrylic plate and mounted under the cover plate for easy fabrication and replacement. The triboelectric PTFE film and bottom electrode (copper foil) were also attached to an acrylic plate and mounted on the base. The top and bottom electrodes had the same thickness (0.022 mm and 0.05 mm, respectively) as those constructed in the TEHTB. Separating the cover plate from the floor tile base required four springs with a spring constant of 29.4 N/mm. When the surfaces of the two different triboelectric layers were pressed into perfect contact, the TEHFT would operate at the highest performance. That was the reason for including the linear vertical guides in the design. Even though it was a prototype, the floor tile design was already intended to be robust, withstanding a high-impact load when humans stepped on it.

### 3.2. Performance Evaluation Method and Experimental Setup

**Figure 8:**
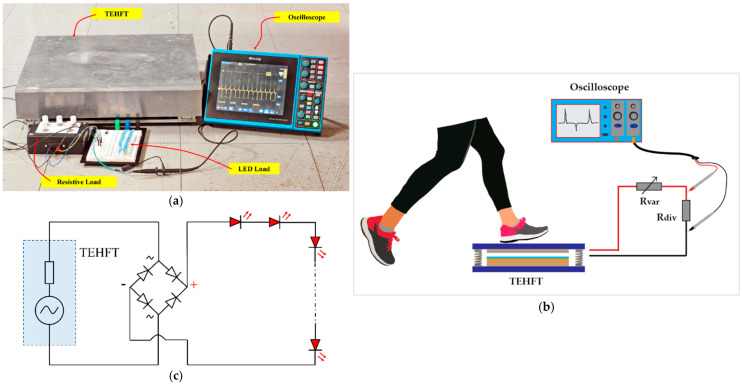
Experiment setup for the TEHFT: (**a**) Laboratory measurement setup; (**b**) the schematic of the experimental setup; and (**c**) schematic of the series LED load connected to the TEHFT.

For performance evaluation, the mechanical input was an impact force from footsteps of a 60-kg heavy human stepping on the floor tile’s cover plate. The output voltage was measured using the same approach as described in the section on TEHTB. The experimental setup is shown in Figure 8a, while a schematic of the experimental setup is depicted in Figure 8b. 

The experimental procedure is as follows.

(1) Measuring the output voltage of the TEHFT equipped with 0.1 mm and 0.2 mm thick PTFE layer.
(2) Connecting an optimal resistive load to the TEHFT and measuring the output voltage, then deriving the accumulative energy at different stepping or pressing frequencies of 0.5, 1, 1.5, and 2 Hz.
(3) Evaluating the real-world performance of the TEHFT with light-emitting diodes (LEDs). The schematic of the experimental setup is shown in Figure 8c.

### 3.3. Experimental Results and Discussion 

#### 3.3.1. Electrical Characteristics of TEHFT with 0.1 mm Thick PTFE Layer

**Figure 9:**
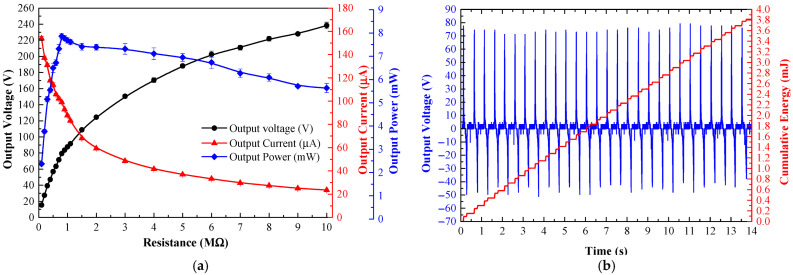
Electrical characteristics of TEHFT with the 0.1 mm PTFE film thickness: (**a**) output voltage, current, and power across resistive loads; and (**b**) graph of output voltage and accumulated energy across the optimal load.

A 60 kg human stepped on the TEHFT producing the cover plate’s RMS acceleration movement of 0.4 g. Twenty peak voltages across a resistive load were measured to calculate the peak current and power output. Figure 9a illustrates the TEHFT’s electrical characteristics. Variable resistive loads ranging from 0.1 MΩ to 10 MΩ were used to determine the optimal harvester load. The optimal load was determined to be 0.8 MΩ with peak voltage, current, and power of 79.28 V, 99.10 µA, and 7.86 mW, respectively. 

A 60 kg subject walked and stepped on the TEHFT at four different stepping frequencies of 0.5, 1, 1.5, and 2 Hz. The stepping on and off was performed for 14 s to fit all data displayed on the oscilloscope screen. Table 1 listed the cumulative energies for those stepping frequencies as 1.01, 1.91, 2.89, and 3.81 mJ, respectively, and the energy per footstep generated by the TEHFT with a 0.1 mm thick PTFE layer was 0.14 mJ/step. The accumulated energy was directly proportional to the number of times that the stepping frequency increased. Figure 9b shows the voltage and cumulative curve at a 2 Hz stepping frequency. The positive voltage was very stable, whereas the negative voltage was not as stable because the stepping-off action was not as consistent as the stepping-on action: the quicker the stepping-off action, the larger the output voltage amplitude. Nevertheless, the peak-to-peak voltage provided by the steps was quite stable at 116.76 ± 1.31 V.

#### 3.3.2. Electrical Characteristics of TEHFT with 0.2 mm Thick PTFE Layer

**Figure 10:**
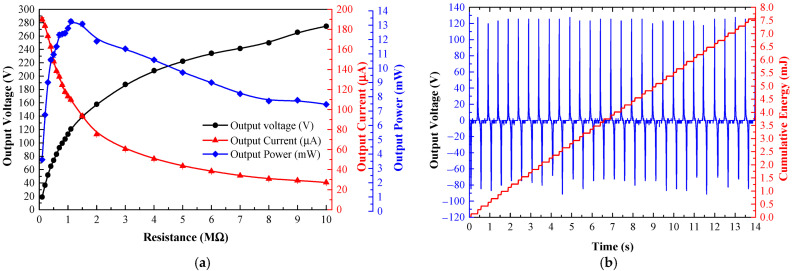
Electrical characteristics of TEHFT with the 0.2 mm PTFE film thickness: (**a**) output voltage, current, and power across resistive loads; and (**b**) graph of output voltage and accumulated energy across the optimal load.

Figure 10a shows the electrical characteristics of the TEHFT across resistive loads from 0.1 MΩ to 10 MΩ. The optimal load for the TEHFT was 1.1 MΩ with peak voltage, current, and power of 120.78 V, 109.80 µA, and 13.26 mW, respectively. 

An optimal load of 1.1 MΩ was connected to the two electrodes of the TEHFT for energy generation calculation. As with the test in the above section, the floor tile’s cover plate was stepped on at four different frequencies of 0.5, 1, 1.5, and 2 Hz. Table 1 listed the total energy for these frequencies as 1.87, 3.64, 5.41, and 7.69 mJ, respectively. The energy per footstep generated by the TEHFT was 0.27 mJ/step. The accumulated energy was directly proportional to the number of times that the stepping frequency increased. Figure 10b displays the voltage and cumulative energy curve provided by 2 Hz stepping frequency, where the peak-to-peak voltage across all steps was higher than that produced by the TEHFT with 0.1 mm thick PTFE layer, at 200.64 ± 2.66 V.

**Figure 11:**
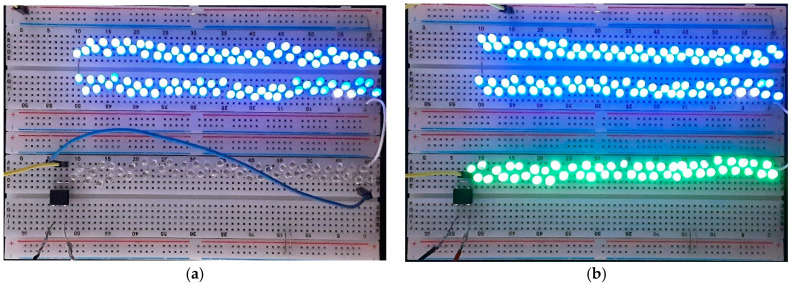
LED load connected to TEHFT: (**a**) TEHFT with 0.1 mm thick PTFE film; (**b**) TEHFT with 0.2 mm thick PTFE film.

The TEHFT with a 0.1 mm thick PTFE layer had a lower optimal load of 0.8 MΩ than the 1.1 MΩ of the TEHFT with a 0.2 mm PTFE layer. This result fully agreed with the findings from Nui et al. [10], who also discovered that as the effective dielectric thickness or cover plate gap width increased, the optimal resistance increased. Nevertheless, the optimal power in their study was found to be constant, while our finding was that the optimal power increased with increasing triboelectric layer thickness and increasing optimal load. The TEHFT with a 0.2 mm thick PTFE layer produced a higher output voltage, current, power, and energy than the one with a 0.1 mm thick PTFE layer. The results matched those of the TEHTB investigation. Both TEHFT with a 0.1 mm and 0.2 mm thick PTFE layer offered sufficiently high power and energy output for powering small electronic devices listed in Table 2. A few footsteps would be needed to generate sufficient energy to supply an MCU + BLE and humidity sensor, while other devices such as temperature sensor, light sensor, and vibration sensor would require fewer steps. To observe energy generation in action, light-emitting diodes (LEDs) were connected to TEHFT with both PTFE film thicknesses of 0.1 mm and 0.2 mm, and the number of connected LEDs was varied. The photos of this setup are shown in Figure 11. For TEHFT with a 0.1 mm thick PTFE layer, the peak voltage across the LEDs was around 211 V, which was sufficient for lighting up 100 LEDs brightly, but they were dimmed when their number was increased to 110 and 120 and could not illuminate 150 LEDs at all. For TEHFT with a 0.2 mm thick PTFE layer, the harvester was able to power up to 150 LEDs at full brightness with an output voltage of 344 V. Although the generated voltage of TEHFT was much higher than the requirements of small electronic devices, the required current would need to be managed by a storage device. Designing a proper energy management system will be our future work.

## 4. Conclusions

This work investigated a contact-separation triboelectric energy harvester. A test bench (TEHTB) and then a prototype TEHFT were designed, constructed, and evaluated. The TEHTB was constructed to validate the design concept and to explore the dependency on triboelectric material thickness, cover plate displacement distance, and cover plate pressing frequency. Proper values of those parameters were then used to design the TEHFT prototype. The excitation frequency of the external force did not affect the output voltage amplitude. However, the cumulative energy increased proportionally with the excitation frequency for TEHTB and TEHTFT. Thinner PTFE film in TEHTB generated more electricity than thicker ones, and 0.1 mm and 0.2 mm thick film performed best in all displacement distance tests, making them the ideal candidate thickness for TEHFT construction. A TEHFT with both 0.1 mm and 0.2 mm PTFE film thicknesses generated sufficient output power and energy for powering small electronic devices and sensor nodes in an automated system. The TEHFT with a 0.2 mm thick PTFE film delivered greater voltage, current, and cumulative energy than that with a 0.1 mm thick PTFE film. Only a few footsteps on the TEHFT, at lower than 2 Hz stepping frequency, of one 60-kg heavy person were needed to generate enough electricity to power 150 LEDs. The contribution of this work to the field of triboelectric energy harvesting is the discovery of the actual parameter values of three most important design parameters for constructing a triboelectric energy-harvesting floor tile that would be applicable to any triboelectric energy harvesting devices. 

## Figures and Tables

**Table 1:** Output voltage, peak power, and cumulative energy across an optimal load for the TEHFT with 0.1 mm and 0.2 mm thick PTFE film at different pressing frequencies.

Stepping Frequency (Hz)	0.1 mm Thickness			0.2 mm Thickness		
	Voltage (V_P-P_)	Peak Power (mW)	Energy (J)	Voltage (V_P-P_)	Peak Power (mW)	Energy (J)
0.5	115.20 ± 2.65	7.36 ± 0.37	1.01 × 10^−3^	199.73 ± 4.10	14.42 ± 0.84	1.87 × 10^−3^
1	113.03 ± 1.07	6.96 ± 0.10	1.91 × 10^−3^	200.59 ± 2.76	14.08 ± 0.13	3.64 × 10^−3^
1.5	113.76 ± 1.24	7.19 ± 0.14	2.89 × 10^−3^	198.83 ± 2.03	13.90 ± 0.18	5.41 × 10^−3^
2	116.76 ± 1.31	7.03 ± 0.19	3.81 × 10^−3^	200.64 ± 2.66	13.96 ± 0.23	7.69 × 10^−3^

**Table 2:** List of small electronic devices that could be powered by the energy generated by TEHFT.

Device	Rated Voltage (V)	Rated Power (W)	Consumption Energy (J)
MCU + BLE [35]	n/a	68 × 10^−5^	6.8 × 10^−3^
Humidity sensor [36]	3.3	99 × 10^−5^	79 × 10^−5^
Temperature sensor [36]	3.3	26 × 10^−6^	5.28 × 10^−9^
Light sensor [36]	3.3	99 × 10^−6^	19.8 × 10^−9^
Vibration sensor [36]	3.3	1.98 × 10^−3^	39.6 × 10^−6^

Note: n/a means not available.
